# O011: Reduction of resistance by sublingual administration of antimicrobials

**DOI:** 10.1186/2047-2994-2-S1-O11

**Published:** 2013-06-20

**Authors:** B Catry, H Laevens

**Affiliations:** 1 Healthcare associated infections (NSIH), Scientific Institute of Public Health, Brussels, Belgium; 2 Eyerbos consulting bvba, Merelbeke, Belgium

## Introduction

Changing an antimicrobial regimen has shown to influence the emergence of antimicrobial resistance, in which the regimen consists of the dose, the treatment interval, the duration of therapy, and the formulation. There is substantial evidence to encourage the use of high dose as short as possible, with a small and regular treatment interval to minimise the risk for the selection of resistant mutants. However, in contrast with these first three aspects of the antimicrobial regimen, little attention is currently paid to 'formulation' in relation to guidelines for a rational antimicrobial therapy to maintain clinical efficacy while reducing the opportunity of resistant strains to have a selective advantage. Formulations of antimicrobial agents have been adjusted in function of the route of administration, which can be local (topical) or systemical. Systemical concentrations of a certain antimicrobial molecule can be achieved either through oral, sublingual, rectal or injectable administration.

## Objectives

The purpose of this work is to explore the different routes of administration with regard to the stimulation of antimicrobial resistance.

## Methods

The one health approach – combining expertise from human and veterinary – will be used throughout the presentation for gathering evidence and exploring potential for the application.

## Results

The author invites the audience to examine the potential manufacturing of sublingual (and rectal) application as alternatives for antimicrobial therapy. Advantages will be explained by terms of correct dosing and timing, compliance and side effects, influence of feed intake on pharmacokinetics (absorption, metabolisation, distribution, elimination), and pharmacokinetic/pharmacodynamic (PK/PD) parameters challenges in commensal and pathogenic bacteria simultaneously.

## Conclusion

Deduced from different observations in human, veterinary medicine and animal studies, reducing the amounts of oral antimicrobial agents might be an underestimated approach to cope with antimicrobial resistant pathogens in both human and veterinary medicine.

## Disclosure of interest

None declared.

